# Enhancing Safety in Clinical Practice: Cross-Sectional, Within-Subjects, Simulated-Use Compliance Study Using BD PosiFlush SafeScrub

**DOI:** 10.2196/78967

**Published:** 2025-12-15

**Authors:** Kyle De Boer, John Simpson, Purnima Sharma, Trevor J Steele

**Affiliations:** 1 Medical Affairs BD Biosciences (United States) Franklin Lakes, NJ United States

**Keywords:** BD PosiFlush SafeScrub, needleless access device disinfection, disinfection compliance, simulation study, scrub-the-hub, scrubbing, flushing

## Abstract

**Background:**

Needleless access devices are essential for intravenous therapy but can be a source of contamination and catheter-related bloodstream infections (CRBSIs) if not disinfected properly. The BD PosiFlush SafeScrub (Becton, Dickinson and Company) is designed to support aseptic nontouch technique (ANTT) by incorporating a built-in reminder to “scrub-the-hub” before flushing. This feature can help improve compliance with disinfection practices and may reduce the risk of microbial contamination.

**Objective:**

This study aimed to evaluate compliance with scrubbing before flushing using the BD PosiFlush SafeScrub in a simulated clinical environment compared with standard disinfection and flushing practices (alcohol swabs and prefilled saline syringes).

**Methods:**

A cross-sectional, within-subjects, simulated-use compliance study was conducted with health care professionals familiarized with BD PosiFlush SafeScrub (a prefilled BD PosiFlush Syringe with an integrated disinfecting unit). Compliance was defined according to the disinfection procedure specified for each scenario; participants were considered compliant with standard practice if they followed their own institutional policy (ranging from 5 to 30 seconds or based on stroke counts), while compliance with BD PosiFlush SafeScrub required scrubbing for at least 10 seconds with a minimum of 8 clockwise and 8 counterclockwise rotations, in accordance with the instructions for use (IFU). Compliance with disinfection was monitored and recorded for both the BD PosiFlush SafeScrub and standard disinfection and flushing practice.

**Results:**

Compliance with catheter hub disinfection was assessed among 60 participants for BD PosiFlush SafeScrub and 57 participants for standard practice. During preaccess procedures (Flush 1), BD PosiFlush SafeScrub achieved 46% compliance versus 21% with standard practice, representing an absolute improvement of 25% and a 119% relative improvement (*P*<.001). During the postmedication procedure (Flush 2), compliance was 22% with BD PosiFlush SafeScrub compared with 13% for standard practice, corresponding to a 9% absolute improvement and 69% relative improvement, although not statistically significant (*P*=.12). Overall, the compliance rate was 34% (81/240 interactions) in the BD PosiFlush SafeScrub group compared with 17% (39/228 interactions) in the standard practice group, representing an absolute improvement of 17% and a relative improvement of 100% (*P*<.001).

**Conclusions:**

The BD PosiFlush SafeScrub, with its integrated disinfection unit, yielded approximately double the scrub-the-hub compliance (34%) before flushing compared to the standard practice of alcohol pads and prefilled saline syringes (17%), supporting its role in facilitating adherence to ANTT, which may reduce microbial growth.

## Introduction

### Background

Vascular access devices (VADs) and needleless access devices (NADs) are essential tools in modern health care, enabling the administration of medications, fluids, and nutrition. NADs—such as needle-free connectors, Y-sites, and stopcocks—are connected to the VAD hub or extension set to support intermittent or continuous infusion. These devices help maintain a closed system, reducing the risk of needlestick injuries and contamination, and are now considered integral to infection control practices [1,2].

Due to their routine use, safe disinfection and flushing practices are critical. Among the most significant complications associated with these devices are catheter-related bloodstream infections (CRBSIs) and central-line-associated bloodstream infections (CLABSIs), which are known to increase patient mortality, hospital length of stay, and health care costs. Recent data show that CLABSIs are associated with a 3.5-fold increased risk of mortality and can extend hospital stays by up to 24.9 days, with associated costs reaching as high as US $90,000 per case [3]. To mitigate these risks, updated guidelines strongly emphasize adherence to standardized insertion bundles, education, and site care and maintenance practices, including the use of antimicrobial NADs and active or passive disinfection [1].

The 2024 revision to the Infusion Therapy Standards of Practice recommends the use of active or passive disinfection to “disinfect the connection surface and sides of the needleless connector attached to any VAD” [1]. In addition, the standard recommends performing active disinfection by vigorous mechanical scrubbing containing 70% isopropyl alcohol or alcohol-based chlorhexidine suitable for use with medical devices. Buetti et al [4], emphasized the importance of hub disinfection using alcohol-based antiseptics for 15 seconds, recommending the use of disinfection caps or active scrubbing techniques to further reduce contamination and biofilm formation. These practices form an essential part of evidence-based bundles aimed at the prevention of CLABSIs.

Despite efforts to educate health care providers and improve disinfection agents, studies indicated that compliance with active disinfection of access ports remains low. A systematic review by Moureau et al [5], found that approximately 50% of central VAD infections stem from bacterial colonization of NADs due to a lapse in aseptic technique and inadequate disinfection. Despite this known risk, adherence to NAD disinfection protocols remains suboptimal, with observed compliance rates as low as 10%. Various factors have been identified that contribute to this noncompliance, including a lack of standardized protocols, high workload and time constraints, forgetfulness, and limited availability of necessary supplies, such as alcohol wipes at the bedside.

Devices like the BD PosiFlush SafeScrub (Becton, Dickinson and Company), which integrates disinfection and flushing into a single-use syringe, offer workflow efficiency and improved adherence to aseptic techniques. These innovations aim to reduce contamination risk and enhance patient safety through simplified, standardized practices. This study sought to evaluate health care providers’ compliance with scrubbing practices when using the BD PosiFlush SafeScrub versus standard methods (alcohol wipes and prefilled syringes) to determine its effectiveness in promoting aseptic vascular access.

### Problem Statement

This study investigated whether the BD PosiFlush SafeScrub can improve compliance with disinfection practice, before flushing compared to standard practice, which may reduce microbial growth.

## Methods

### Study Design

This cross-sectional, within-subjects, simulated-use compliance study aimed to evaluate compliance with disinfection protocols using the BD PosiFlush SafeScrub compared with BD PosiFlush prefilled saline syringes with alcohol swabs. “BD PosiFlush saline syringe with alcohol swabs” is hereafter referred to as the “standard practice.” Sixty registered nurses from varied clinical specialties, including emergency department, intensive care unit (ICU), critical care unit, outpatient infusion, home health care, and medical-surgical units, were enrolled from acute care facilities. The recruitment and scheduling of participants for this simulation, survey-based study was done in a usability lab setting at Delve (Delve Research), a design consultancy and research services provider. The study was conducted in two geographical locations, Delve’s Philadelphia office and Boston office, from June to July 2024.

### Study Participants

All the study participants were healthy individuals. The registered nurses were from the emergency room, ICU, critical care unit, outpatient infusion center, home health care, and medical-surgical units. No participant had any hand injuries, latex sensitivity, or other conditions that might affect scrubbing behavior. The participants recruited for the study had prior experience with the care and maintenance of VADs and NADs.

Participants were recruited from acute care facilities with more than 50 beds. Inclusion criteria required participants to have experience with VADs and disinfection practices. Eligible individuals needed to be at least 18 years of age and proficient in understanding written and spoken English. Additionally, participants were required to have a minimum of 2 years of related nursing experience. They must not have participated in any usability studies related to flushing with prefilled syringes within 6 months of their participation in this study. This structured approach ensured that the study included a diverse and experienced group of health care professionals, providing a comprehensive evaluation of the BD PosiFlush SafeScrub in various clinical scenarios.

### Materials

The materials used in this study included the BD PosiFlush SafeScrub Prefilled Saline Flush Syringe (Becton Dickinson and Co), standard alcohol swabs, and the BD PosiFlush Prefilled Flush Syringe (Becton Dickinson and Co). A mannequin was positioned in a simulated hospital bed or stretcher, with one arm outfitted with a peripherally inserted central catheter (PICC; Becton Dickinson and Co) and the other with a peripheral intravenous catheter (PIVC; Becton Dickinson & Co). The study consisted of 60 simulated-use sessions, each lasting 60 minutes. Participants were observed through four simulated push medication scenarios, using both the standard practices and the BD PosiFlush SafeScrub. They were provided with all necessary materials typically found in a clinical setting, including gloves, alcohol swabs with 70% isopropyl alcohol, flush syringes filled with saline, prefilled simulated medication syringes (filled with colored saline), and other ancillary components. All participants were asked, before beginning the study, if the environment was similar to what they would see in their clinical areas of practice. This was done to ensure that the way they would respond to the scenario would be very close to what would occur in a real clinical setting.

**Figure 1:**
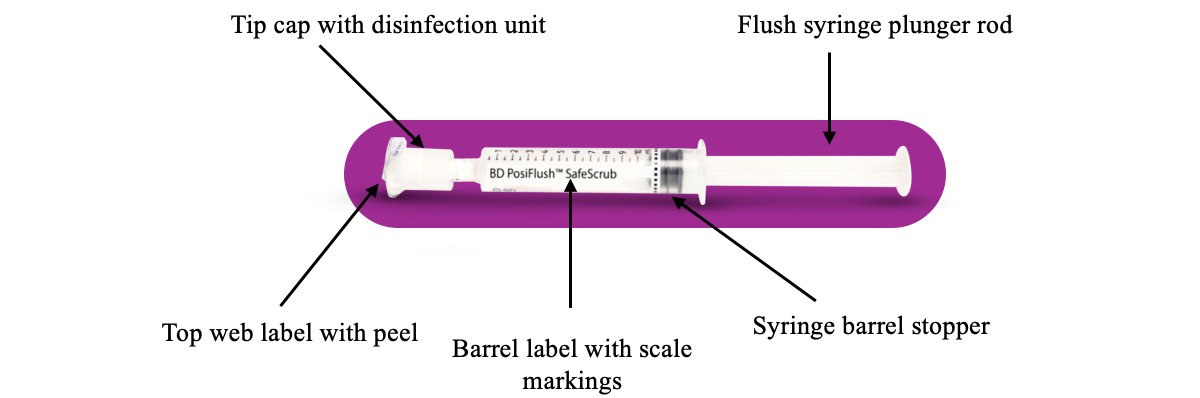
BD PosiFlush SafeScrub labeled image.

The BD PosiFlush SafeScrub combines a prefilled saline syringe with an integrated disinfection cap, simplifying and streamlining the scrubbing and flushing workflow (Figure 1). It is a device designed to improve the disinfection process of NADs in vascular access systems. The dual-purpose design aims to simplify the disinfection process and improve compliance among health care providers with an all-in-one scrub and flush solution, ensuring consistent and effective disinfection before each flush. As a single-use product, it minimizes the risk of cross-contamination, making it a valuable tool in promoting aseptic nontouch technique (ANTT) in clinical settings [1]. By supporting consistent and effective disinfection, such devices play a vital role in reducing the contaminants that can cause bloodstream infections and enhance overall patient care.

### Study Protocol

Following consent and study enrollment, participants were informed that their hospital adopted a new catheter hub disinfection product. They were then asked, “What would you do?” Based upon their responses, participants either received the instructions for use (IFU) or were shown a demonstration video (along with the IFU), which were the only 2 training formats provided to ensure consistency. A total of 60 simulated-use sessions, each lasting approximately 60 minutes in duration, were conducted. They were instructed to follow aseptic technique throughout the medication administration process to minimize the potential for touch contamination. Each participant completed 4 simulated intravenous push medication scenarios, 2 using the standard practice and 2 using the BD PosiFlush SafeScrub. The order for using each syringe and scenario was randomized. The randomization determined both the sequence of device use (standard practice vs SafeScrub) and the catheter type (peripherally inserted central catheter [PICC] vs peripheral intravenous catheter [PIVC]) for each scenario. A 4-factorial forced randomization design was used. Participants were randomly assigned to one of the several pregenerated scenario sequences using a computer-based randomization algorithm. Each participant received their assigned sequence upon enrollment, ensuring a balanced distribution of scenario order and device type.

Each medication administration scenario included a flush before medication delivery and a flush postdelivery; however, the medication delivery step was solely for contextual purposes and not included in data analysis. The data were collected on compliance with scrubbing before flushing within the medication administration process. Throughout the study, the focus was on the scrubbing and flushing process, comparing the standard practice with that of the BD PosiFlush SafeScrub.

Three pilot studies were conducted to identify any potential issues and provide the most realistic setup and scenarios for the current study. The protocol, which was chosen after the pilot studies, included nonemergent clinical scenarios and no planned distractions for the participants. This protocol was then applied to the full data collection for all 60 participants.

These panels illustrate the simulated clinical environments developed for evaluating catheter hub disinfection practices. In each setup, the disinfection site (hub) is depicted to show the scrub-the-hub step performed before flushing. Incorporating both PICC and PIVC scenarios allowed the study design to reflect routine vascular access procedures and provided a standardized framework for assessing compliance with hub disinfection across intervention and control groups.

**Figure 2:**
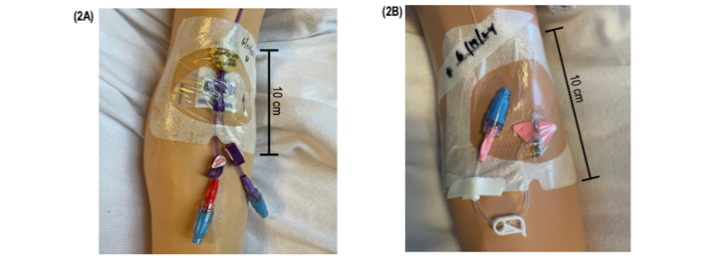
Simulated catheter set-ups demonstrating scrub-the-hub disinfection workflow. (A) Peripherally inserted central catheters. (B) Peripherally intravenous catheter.

A study session was set up as follows, a mannequin was positioned in a simulated hospital bed or stretcher, with one arm outfitted with a PICC and the other with a PIVC. Both catheters were equipped with NADs. The arrangement and location of the PICC line setup and the PIVC line setup on the mannequin are shown in Figure 2A and 2B, respectively. These images accurately represent a clinical PICC or PIVC setup, with both lines connected to a NAD. The NAD was not covered with a passive disinfecting cap, reflecting certain clinician practices that may not require scrubbing before accessing the NAD after a passive cap has been removed.

The study protocol included several key steps outlined as follows:

- Flush 1: this was the intended scrub and flush before accessing the line for the medication delivery. It ensured that the line was free of occlusion before medication was delivered.
- Medication delivery: after Flush 1, the medication was administered. It is important to note that there was no compliance scoring for the scrubbing done immediately before this step.
- Flush 2: after the medication was delivered, there was a second scrub to disinfect the hub, followed by a flush step. This was intended to disinfect the hub before a final flush, which ensures no medication residue remains in the line.
- Compliance scoring: participants were evaluated based on their adherence to the protocols for Flush 1 and Flush 2. Absolute improvement in compliance was calculated as the difference in compliance rates between the BD PosiFlush SafeScrub group and the standard practice group, {(absolute improvement) = [(compliance of SafeScrub) – (compliance of standard practice)]}. Relative improvement in compliance was calculated as the absolute improvement expressed as a percentage of the standard practice compliance rate, {[relative improvement] = [(compliance SafeScrub) – (compliance standard) / (compliance standard)] times 100]}.

In summary, the protocol is designed to ensure proper disinfection or cleaning during the administration of flushing and medication administration via an intravenous line, with specific compliance measures for the disinfecting steps before and after the medication delivery. The study compared the standard practice with the BD PosiFlush SafeScrub.

### Data Collection

Data collected during the simulation study included user demographics, current practices, and hospital policies on scrubbing and flushing. Specific metrics recorded were the frequency and duration of scrubbing before flushing for both the standard practice and BD PosiFlush SafeScrub, as well as the drying time allowed before accessing the NAD. For each location, a facilitator was in the room with the participant, and note takers and observers viewed from a separate room or remotely by way of streaming video or poststudy video review (Multimedia Appendix 1). Participants’ overall impressions and reactions to each method were also collected. Data on compliance was collected through direct observation and video recording of the disinfection and flushing workflow process. For standard practice scenarios, participants were asked what their hospital policy was for scrubbing prior to flushing, and they were considered “compliant” if they followed their own individual hospital policy. Policies ranged from scrubbing 5-30 seconds, and some involved the number of times scrubbing back and forth rather than duration. For BD PosiFlush SafeScrub scenarios, participants were considered “compliant” if they scrubbed for at least 10 seconds, for a minimum of eight repetitions clockwise alternating with eight repetitions counterclockwise, in accordance with the IFU. The time was recorded using a stopwatch on a phone. We acknowledge that defining compliance according to the BD PosiFlush SafeScrub IFU (ie, ≥ 10-second scrub with at least eight clockwise and eight counterclockwise repetitions) while using institution-specific scrubbing policies for the standard practice arm introduces methodological asymmetry. However, this approach was intentionally chosen to preserve external validity and generalizability by reflecting the diverse, real-world practices of health care providers in different institutional contexts. In pragmatic study designs, such variation in comparator definitions is often embraced to capture how an intervention performs under routine operational conditions without artificially standardizing comparator practices [6,7].

Moreover, our goal was to assess whether the SafeScrub device promotes compliance beyond prevailing norms, rather than to apply a uniform compliance threshold across both conditions. By allowing participants to follow their own institutional standards in the comparator arm, we both minimized bias from altering habitual workflows and maintained the relevance of our findings to everyday practice.

Thus, the differing compliance definitions enhance the external validity of our results and reinforce the practical utility of the BD PosiFlush SafeScrub device within diverse clinical settings.

Additional data collection methods included prestudy screener criteria, manual observations by note takers, video recordings and images for poststudy analysis, and reviews by data analysts to finalize results and uncover additional insights. After completing all 4 scenarios, participants were asked about their hospital policies and what they stated regarding scrubbing catheter hubs. After participants used the BD PosiFlush SafeScrub in the study, they were asked to complete a user acceptance survey, which was comprised of 15 questions. The survey incorporated both 1-5 and 0-10 Likert-type scales, intentionally selected based on the nature of the information being gathered. The 1-5 scale was used for most survey items to assess agreement with specific usability and compliance statements related to the device (eg, ease of use, learning curve, and reinforcement of guidelines). This scale is standard for capturing general attitudes and perceptions and satisfaction levels with sufficient sensitivity while minimizing respondent fatigue. The 0-10 scale was selectively applied to 2 questions that evaluated broader behavioral intentions–likelihood to recommend and inclination to use the device in clinical practice. These items benefit from greater granularity and are commonly used in health care research. The expanded range enables differentiation among high-positive responses (eg, 8 vs 10), which is important for assessing clinical acceptability and forecasting real-world implementation.

To aid interpretability and comparison across all survey items, responses were summarized using both average ratings (mean [SD]) and the percentage of respondents selecting a high response (defined as ≥4 for 1-5 scale items and ≥8 for 0-10 scale items).

Prior to enrollment, we conducted a priori power analysis based on the three pilot studies for a 2-tailed independent samples *t* test to determine the required sample size, targeting 80% power at α=.05 to detect a meaningful between-group effect.

### Data Analysis

Data were analyzed using R version 4.3.0 (R Foundation for Statistical Computing). Descriptive statistics summarize the data, and inferential statistics, such as chi-square tests, were used to determine the significance of the differences observed. The results were considered significant for all analyses at a 5% level of significance (*P*<.05).

### Ethical Considerations

No patient data were collected in this study, as patients were not included. In accordance with the ethical standards of the Declaration of Helsinki, written informed consent was obtained from all study participants before study initiation [8,9]. Participants were assured that all identifying information would remain confidential and would not be disclosed in any study-related publications. All data were deidentified before analysis to maintain participant anonymity.

Participants were informed of the potential risks associated with the procedure. Furthermore, the intervention involved a commercially available, marketed product containing substances already used in routine clinical practice, specifically isopropyl alcohol and 0.9% sodium chloride (normal saline). Although formal IRB approval was not required due to the nature of the simulation and the minimal risk posed to participants who were only exposed to the test device and preinserted catheters, this simulated use study was conducted in accordance with clinical and ethical best practices under the guidance of Delve (Delve Research).

## Results

### Study Participants

Sixty registered nurses participated in the study, representing a diverse clinical background and varying levels of professional experience. The demographics of the participants are shown in Table 1. All participants completed the simulation and provided complete demographic and clinical information. Participants were employed across multiple health care settings, including adult emergency departments (n=11), adult intensive care or critical care units (n=16), outpatient infusion centers (n=15), home health care (n=5), and medical-surgical units (n=13). The average overall clinical experience among participants was 15.87 years, with an average of 9.09 years of experience in their current primary department.

**Table 1:** Demographic and clinical background information of participants.

Participants number (N=60)			Variables
**Primary department of occupation, n**			
	Adult emergency room nurse	11	
	Adult critical care nurse	16	
	Outpatient infusion center nurse	15	
	Home health care (HHC) nurse	5	
	Med-surg nurse	13	
Overall years of experience (years), mean (SD)			15.87 (10.23)
Years of experience in the current primary department (years), mean (SD)			9.09 (7.17)

### Hospital Policy for Scrubbing Time

Participants reported a wide range of institutional policies related to catheter hub disinfection practices before flushing. Details on hospital policy counts and ranges are presented in Table 2. The most cited policy specified a scrubbing duration of 10 seconds, reported by 22 participants. A smaller number reported (n=9) scrubbing durations of 15 seconds, 15-30 seconds, and 20-30 seconds. One participant each reported scrubbing durations of 5-10 seconds, 5-15 seconds, or 25-30 seconds.

Variations in policy also included combined time and technique directives. Two participants noted a requirement of 10 scrubbing rotations, and one specified 10 rotations using 2 separate swabs. One participant reported different time-based policies for peripheral versus central lines (15 and 30 seconds, respectively), and another reported a general scrub for peripheral lines with a specified 15-second scrub for central lines. Three participants reported not knowing their hospital disinfection policy, with one stating that no disinfection policy was in place. A few participants (n=4) indicated policies in which only the initial flush required scrubbing, with no scrubbing required after medication administration.

**Table 2:** Reported hospital policies and ranges.

Reported hospital policy	Participants with hospital policy, n (%)
3-5 seconds first flush, no scrubbing second flush	1 (1.6)
5 seconds scrubbing time	1 (1.6)
10 seconds scrubbing time	22 (36.6)
15 seconds scrubbing time	9 (15)
15 seconds first flush, no scrubbing second flush	2 (3.3)
10 seconds first flush, no scrubbing second flush	1 (1.6)
30 seconds scrubbing time	6 (10)
5-10 seconds scrubbing time	1 (1.6)
5-15 seconds scrubbing time	1 (1.6)
10-15 seconds scrubbing time	2 (3.3)
15-30 seconds scrubbing time	2 (3.3)
25-30 seconds scrubbing time	1 (1.6)
20-30 seconds scrubbing time	2 (3.3)
10 rotations scrubbing time	2 (3.3)
10 rotations, 2 swabs	1 (1.6)
Peripheral just scrub, no time requirement Central 15 seconds	1 (1.6)
15 seconds peripheral, 30 seconds central	1 (1.6)
No requirement – as stated by the participant	1 (1.6)
Does not know – participants not familial with hospital policy	3 (5)

### Compliance

Compliance with catheter hub disinfection protocols was assessed during both preaccess (Flush 1) and postmedication (Flush 2) procedures using either standard practices or the BD PosiFlush SafeScrub. For the BD PosiFlush SafeScrub scenarios, compliance was defined as scrubbing for ≥ 10 seconds with a minimum of 8 clockwise and 8 counterclockwise repetitions, as per the IFU. A total of 60 participants were evaluated in the SafeScrub group. In contrast, data from 57 participants were analyzed for standard practice due to incomplete hospital policy reporting from three individuals, rendering their standard practice compliance scoring invalid. Table 3 summarizes the compliance data for the participants using the standard practice and BD PosiFlush SafeScrub. Compliance was assessed across both the Flush 1 (preaccess) and Flush 2 (postmedication) procedures, and the total number of compliant interactions (overall compliance) was calculated for each method.

**Table 3:** Compliance data for the participants using the standard practice and BD PosiFlush SafeScrub.

Category			Standard practice (N=57)		BD PosiFlush SafeScrub (N=60)
**Flush 1 (preaccess)**					
	PICC^a^, n (%)	13 (23)		27 (45)	
	PIVC^b^, n (%)	11 (19)		28 (47)	
	Total, n/N (%)	24/114 (21)		55/120 (46)	
**Flush 2 (postmedication)**					
	PICC, n (%)	6 (11)		12 (20)	
	PIVC, n (%)	9 (16)		14 (23)	
	Total, n/N (%)	15/114 (13)		26/120 (22)	
	Overall compliance, n/N (%)	39/228 (17)		81/240 (34)	
	*P* value	—^c^		<.001	

^a^PICC: peripherally inserted central catheter.

^b^PIVC: peripheral intravenous catheter.

^c^Not available.

### Flush 1 (Preaccess)

The BD PosiFlush SafeScrub achieved a compliance rate of 46% (55/120), significantly higher than the 21% (24/114) observed with standard practice. This corresponds to an absolute improvement of 25% (*P*<.001) and a relative improvement of 119% in preaccess disinfection compliance when using the BD PosiFlush SafeScrub.

### Flush 2 (Postmedication)

Post-medication compliance was also higher with BD PosiFlush SafeScrub, at 22% (26/120) interactions, compared to just 13% (15/114) interactions with the standard practice. This represents an absolute improvement of 9% and a relative improvement of 69%; however, the difference did not reach statistical significance (*P*=.12).

### Overall Compliance

Across all interactions, the overall compliance rate was 34% (81/240) interactions in the BD PosiFlush SafeScrub group compared with 17% (39/228) interactions in the standard practice group, representing an absolute improvement of 17% and a relative improvement of 100% (*P* <.001).

An exploratory post-hoc analysis applying a 10-second threshold in participants using the standard practice and BD PosiFlush SafeScrub was also conducted. A 2-sample proportion test demonstrated a statistically significant difference between groups (*P*<.000) such that the BD PosiFlush SafeScrub group had a higher overall compliance when compared with the standard practice group.

### Summary of Root Causes

Participants often thought that they followed the IFU for scrubbing the BD PosiFlush SafeScrub for at least 10 seconds, for a minimum of 8 repetitions clockwise alternating with 8 repetitions counterclockwise, and many noted that they were counting times back and forth rather than the seconds in their head. For BD PosiFlush SafeScrub and standard practice, most participants reported one of the following reasons for not following hospital policy or IFU.

- The participants believed that they were following their hospital policy.
- The participant reported that they knew they were in a study and that no actual patient would be at risk for infection if they did not scrub the entire required time (study artifact).
- The participant reported that clinicians generally do not follow the hospital policy for scrubbing.

### User Acceptance Survey Results

The results of the user acceptance survey revealed consistently high levels of agreement with positive usability statements, indicating acceptance of the device within clinical practice. The user acceptance survey responses are summarized in Table 4.

**Table 4:** User acceptance survey results for the BD PosiFlush SafeScrub device.

Parameters		Agreement (respondents selecting ≥4 on 1-5 scale or ≥8 on 0-10 scale), %	Average rating, mean (SD)
**SafeScrub Device (1-5 rating)**			
	Ease of use	96.6	4.6 (0.62)
	Learning curve	96.6	4.7 (0.58)
	Impact on patient safety	91.6	4.6 (0.64)
	Compliance with best practices	88.3	4.4 (0.75)
	Reinforcement of guidelines	96.6	4.7 (0.52)
	Ease of compliance	93.3	4.6 (0.55)
	Labeling and integration impact	85	4.5 (0.62)
	Efficiency improvement	58.3	4.4 (0.83)
	Frequency of distractions	86	3.1 (0.98)
	Impact of distractions	85	4.1 (0.76)
	Overall sentiment	91.6	4.4 (0.67)
**Clinical acceptability/impact (0-10 rating)**			
	Inclination to use in practice	43.3 (≥8 on scale)	8.7 (1.93)
	Likelihood to recommend	78.3 (≥8 on scale)	8.5 (1.75)

For ease of use and learning curve, a total of 96.6% of participants agreed that the BD PosiFlush SafeScrub device is easy to use, with an average rating of 4.6 on a 5-point scale. Similarly, 96.6% indicated that most clinicians would learn to use the device quickly, yielding an average learning curve rating of 4.7. These findings reflect minimal training burden and favorable ergonomics.

For the impact of safety and compliance regarding patient safety, 91.6% of respondents reported that the device contributed to reducing the risk of microbial growth and potential CRBSIs, with an average rating of 4.6. In terms of clinical compliance, 88.3% believed that the device improves adherence to evidence-based best practices—such as ANTT and “scrub-the-hub”—relative to their current practice, with a rating of 4.4. Furthermore, 96.6% reported that the device design reinforces compliance with “scrub-the-hub” guidelines (rating: 4.7), and 93.3% indicated that becoming compliant with its use would be easy (rating: 4.6).

Device labeling and integration features, including the peel tab and built-in disinfection unit, were positively received by 85% of participants, with an average rating of 4.5. A total of 58.3% of participants felt that the elimination of the need to search for alcohol wipes improved workflow efficiency (rating: 4.4), and 43.3% of participants indicated a high inclination to adopt the device in daily practice, with a mean rating of 8.7 on a 10-point scale. Upon further investigation, we observed that around 30% did not select a response, leading to a low inclination for adoption of the device. Notably, 86% of respondents acknowledged frequent distractions during catheter access tasks, and 85% agreed that such distractions may cause clinicians to rush the disinfection process, both rated at 4.1.

The overall sentiment toward the BD PosiFlush SafeScrub was favorable, with 91.6% of participants rating their experience 4 or above on a 5-point scale (mean 4.4, SD 0.67). Additionally, 78.3% expressed a high likelihood of recommending the device to colleagues, with a recommendation score of 8.5 out of 10.

These findings suggest that the BD PosiFlush SafeScrub is perceived as a user-friendly and effective tool that enhances compliance with infection prevention protocols and supports safer, more effective clinical workflows.

## Discussion

### Principal Findings

The purpose of this study was to evaluate compliance with scrubbing before flushing using the BD PosiFlush SafeScrub in a simulated clinical environment compared with standard disinfection and flushing practices (alcohol swabs and prefilled saline syringes). The results revealed that the BD PosiFlush SafeScrub, with its integrated disinfection unit, yielded approximately double the scrub-the-hub compliance (34%) before flushing compared to the standard practice of alcohol pads and prefilled saline syringes (17%). This improvement aligns with prior compliance-focused interventions. In adult acute care, Samanta et al [10] demonstrated that structured interventions, including daily audits, bedside teaching, and reinforced scrub-the-hub protocols, raised compliance rates for insertion checklists (83.3%), full barrier precautions (78.4%), and scrub-the-hub technique (65%), underscoring the value of both behavioral and system-based measures in high-risk environments [10]. A recent review reports that the Institute for Safe Medication Practices suggests that educational interventions are the easiest to implement, but they are the least effective approaches [11]. Further, the authors stated that the “‘forcing functions,’ which are safety design features that necessitate actions occurring in the same way each time, are the most eﬀective type of intervention, as they can eliminate the risk of error (ie, a high leverage strategy based on system reliability), although they are more complex to introduce.”

Bundle approaches have also been investigated to improve patient safety. Devrim et al [12], reported that implementation of a central line bundle—including split-septum devices, needleless connectors, and single-use prefilled flushing syringes—reduced CLABSI incidence in pediatric oncology patients by 59% with an estimated cost saving of approximately US $208,977 [12]. Similarly, Samanta et al [10], observed a 43% reduction in CLABSI rates in an adult polytrauma ICU, with 49 consecutive CLABSI-free days, reinforcing the link between compliance and infection prevention. In a neonatal ICU, another study demonstrated that routine scrubbing of catheter hubs reduced CLABSI incidence from 1.89 to 0.23 per 1000 central line days [13]. Collectively, these findings confirm that interventions ranging from bedside timing aids to institutional bundles and device-integrated solutions can deliver both clinical and economic benefits across diverse populations.

Compared with system-level programs that rely on continuous auditing and retraining, the BD PosiFlush SafeScrub offers a device-level behavioral nudge that integrates disinfection into the flushing step. By reducing reliance on memory, product availability, and accurate time estimation—factors vulnerable to erosion under workload stress—the device helps standardize practice in real-world, high-pressure environments such as emergency departments or ICUs. Its integrated design not only simplifies workflow but also encourages habit formation and consistent adherence, even when time is constrained.

These findings align with another study, which showed that implementing a universal disinfection cap program reduced CLABSI rates by over 40% with annual cost savings of US $282,840, reinforcing the association between disinfection compliance and economic benefits [14]. By embedding disinfection directly into practice, BD PosiFlush SafeScrub provides a low-barrier complement to larger institutional initiatives, helping to close the persistent gap between policy awareness and actual compliance.

According to the Infusion Nurses Society Infusion Therapy Standards of Practice (Ninth Edition, 2024), disinfection of the needleless connector is required before each access, including before flushing and locking after medication administration [1]. While this study found that the BD PosiFlush SafeScrub device improved preflush compliance substantially, the same effect was not observed following administration of medication (Flush 2 compliance). This endpoint was powered a priori to detect such differences, suggesting that improvements in post-medication scrub compliance may require interventions beyond device integration.

In addition, participant feedback further highlighted variability in adherence. Several participants believed they were following the BD PosiFlush SafeScrub IFU (at least 10 seconds with a minimum of 8 clockwise and eight counterclockwise repetitions) but admitted to counting motions rather than measuring time. In standard practice scenarios, deviations were often attributed to perceived protocol familiarity, low perceived risk during study participation, or inconsistent hospital policies ranging from 3 to 30 seconds. These observations emphasize the challenges of ensuring consistent adherence to disinfection protocols and reinforce the need for education, standardization, and behavioral support tools [11].

### Impact Statement

This study demonstrated a clinically significant increase in disinfection compliance following the use of BD PosiFlush SafeScrub. Enhanced compliance with disinfection practices is critical for helping to reduce the microbial burden, which may lead to health care–associated infections, providing better health care, and lowering associated costs. By promoting a culture of accountability and standardizing device disinfection, this intervention could serve as a model for improving patient safety and quality of care in various health care settings.

### Limitations of the Study

This study has several limitations. The simulated nature of the design, while valuable for the controlled exploration of device performance, cannot fully capture the complexities, time pressures, and interruptions present in real-world clinical environments across different health care settings. Simulated conditions may inadvertently inflate compliance rates due to factors such as the Hawthorne effect, simplification of tasks, scripted scenarios, and the absence of real patient risk, which could alter participant behavior. For instance, clinicians might demonstrate more meticulous disinfection in a simulation when no actual patient is in danger. These limitations affect ecological validity and underscore the need for further research to validate these findings under actual clinical conditions, where time pressures, competing priorities, and workflow disruptions are present.

In addition, the generalizability of results may be limited due to all participants being based in the United States. Further, not all aspects of compliance and disinfection were measured, leaving some critical elements unevaluated, and compliance improvement was only assessed over a limited time frame, preventing conclusions about long-term adherence. Another limitation is that the intervention group received structured training (via IFU or demonstration video), whereas the control group followed existing hospital procedures without equivalent refresher training. This methodological asymmetry introduces a confounding effect, as training alone can increase adherence regardless of device performance. Further studies should consider designs that isolate training effects, such as crossover trials or parallel group designs, to better determine device-specific impact.

Additionally, given the current health care environment, cost-conscious decision-making, and prevailing standard practices, we recognize that the unit cost of an alcohol swab and a prefilled flush may be lower than that of the study product, BD PosiFlush SafeScrub. However, the true value of the device lies in its ability to enhance compliance, as demonstrated in this study. Its integrated design serves as a visual and functional prompt for clinicians to scrub the access site prior to flushing, helping reduce the risk of introducing contaminants that could lead to CRBSIs. Further studies should therefore evaluate cost-effectiveness, implementation feasibility, and sustainability dimensions in real-world settings.

Finally, three pilot studies informed the main study design. Based on the clinician’s feedback, the control group was evaluated against their own hospital policies rather than a ≥10-second threshold to preserve external validity by reflecting real-world practice. We acknowledge that this approach may introduce methodological asymmetry. To address this concern, we conducted an exploratory post hoc analysis applying a 10-second compliance threshold to both groups. This analysis demonstrated a statistically significant difference in favor of the intervention, indicating that the findings are robust regardless of the compliance definition applied.

### Conclusions

The BD PosiFlush SafeScrub device approximately doubled the scrub-the-hub compliance before flushing compared to alcohol pads and prefilled saline syringes, simplifying workflow by embedding disinfection into the flushing step. Further real-world studies are needed to confirm cost-effectiveness and environmental impact across diverse health care settings.

## Appendix

Multimedia Appendix 1 Simulated room set-up and Illustration of BD PosiFlush SafeScrub Prefilled Saline Syringe scrubbing technique.

## Abbreviations

ANTT: aseptic nontouch technique
CLABSI: central-line-associated bloodstream infection
CRBSI: catheter-related bloodstream infection
ICU: intensive care unit
IFU: instructions for use
NAD: needleless access device
PICC: peripherally inserted central catheter
PIVC: peripheral intravenous catheter
VAD: vascular access device
